# *MUC5AC* Upstream Complex Repetitive Region Length Polymorphisms Are Associated with Susceptibility and Clinical Stage of Gastric Cancer

**DOI:** 10.1371/journal.pone.0098327

**Published:** 2014-06-02

**Authors:** Chenghua Wang, Jinshen Wang, Yiqing Liu, Xueliang Guo, Chunqing Zhang

**Affiliations:** 1 Department of Emergency Center, Shandong Provincial Hospital affiliated to Shandong University, Jinan, Shandong, China; 2 Department of General Surgery, Shandong Provincial Hospital affiliated to Shandong University, Jinan, Shandong, China; 3 Department of Laboratory Medicine, Shandong Provincial Hospital affiliated to Shandong University, Jinan, Shandong, China; 4 Cystic Fibrosis/Pulmonary Research and Treatment Center, University of North Carolina at Chapel Hill, Chapel Hill, North Carolina, United States of America; 5 Department of Gastroenterology, Shandong Provincial Hospital affiliated to Shandong University, Jinan, Shandong, China; University of Navarra, Spain

## Abstract

MUC5AC was deemed to be involved in gastric carcinogenesis since aberrant MUC5AC expression has been repeatedly detected in patients with gastric cancer (GC). In this study, length polymorphisms in a complicated repetitive region adjacent to *MUC5AC* promoter were assessed in 230 patients with GC and 328 cancer-free controls. Alleles of 1.4 and 1.8 kb were significantly more prevalent in GC group than in controls. In contrast, 2.3 and 2.8 kb alleles occurred at significantly lower frequencies in patients than in controls. Alleles were then classified into susceptible (S; 1.4 and 1.8 kb), protective (P; 2.3 and 2.8 kb) and null (N; all other alleles) categories with respect to their linkage with the susceptibility to GC. Individuals with genotype SS had a 2.7-fold increased risk of GC occurrence, but PN genotype was associated with a significantly reduced risk of this cancer. Moreover, homozygous or heterozygous individuals with one or two copies of 1.4 kb allele showed an earlier age of onset and more advanced metastasis stage compared with patients without this allele (Bonferroni corrected p = 1.35×10^−4^ and 6.60×10^−4^ accordingly), whereas homozygous patients with two copies of 1.8 kb allele were linked to less advanced GC TNM stage. Our results suggest that certain genetic variations in *MUC5AC* upstream repetitive region are associated with the susceptibility and progression of GC.

## Introduction

Gastric cancer (GC) is one of the most common malignancies and the second leading cause of cancer-related death worldwide [1]. However, its mechanism remains unclear. Although some environmental factors, such as diet, cigarette smoking and *Helicobacter pylori*, may contribute to carcinogenesis of gastric epithelial cells [2]–[4], only a fraction of the population exposed to such risk factors develop GC during their lifetime. This suggests that genetic factors play a crucial role in determining an individual's susceptibility to GC [5], [6].

Mucins are a group of diverse, complex, highly glycosylated extracellular proteins important in maintaining epithelial homeostasis. Cancer cells are often observed to express aberrant forms or amounts of mucins, and these aberrations are thought to play a role in carcinogenesis, especially in regulation of tumor cell differentiation, proliferation, and tumor invasion [7]. For example, overexpression of MUC1 and MUC4 in several different forms of adenocarcinoma contributed to the regulation of cancer cell proliferation via an interaction with epidermal growth factor regulator (EGFR) and extracellular signal-regulated kinases [8]. Velcich *et al*. demonstrated that *Muc2* ^−/−^ mice develop adenomas in the intestine that progress to invasive adenocarcinomas [9], suggesting a protective role for MUC2 in intestinal tumorigenesis.

MUC5AC is a secreted gel-forming mucin and a marker of gastric foveolar epithelial cells [10]. MUC5AC was deemed to be involved in gastric carcinogenesis since gastric carcinoma was found to contain a lower level of MUC5AC expression than normal gastric mucosa [11]–[13], and several clinical studies demonstrated that MUC5AC expression level was associated with severity of GC; however, these data were inconsistent [12], [14]. There has been little research on MUC5AC function and the mechanisms underlying its role in GC development, until recently it was reported that silencing MUC5AC, using a small hairpin RNA-containing lentivirus, increased gastric cancer cell invasion and migration in vitro [13]. This adds to evidence that altered levels of MUC5AC expression may be involved in GC pathogenesis. Functional genetic polymorphisms in the regulation region may affect *MUC5AC* gene expression and then contribute to an individuals' susceptibility to gastric cancer.

Repetitive regions of DNA are common throughout the human genome and are characterized by their dynamic, unstable features [15], [16]. They are the major generator of genetic variation and are considered to underlie substantial genetic variability, with novel mutations in such regions explaining much of the ‘missing’ heritability in polygenic diseases [17], [18], including GC [19]. However, this kind of genetic variation cannot be included in genome-wide association studies (GWAS) panels and is challenging to assess reliably. Intensive review of the *MUC5AC* upstream regulation region and around identified a complicated repetitive region (termed *MUC5AC-u* repetitive region). We undertook this case-control study to determine the nature and extent of genetic polymorphisms within this region, and to explore the association of each genetic variant with the occurrence and progression of GC.

## Methods

### Database searches and analysis of the upstream region of *MUC5AC*

The UCSC genome browser (http://genome.ucsc.edu) and the GRCh37/hg19 release of the human genome were used to generate a map showing the location and major genomic features of the *MUC5AC* gene, including histone H3 lysine 27 acetylation (H3K27AC) status, transcription factor binding sites, common single-nucleotide polymorphisms (SNPs), and repetitive sequence genomic features of the upstream region. The DNA sequence of the upstream region was downloaded from the Ensembl (http://useast.ensembl.org/Homo_sapiens/ Info/Index).

### Ethics statement

This study was conducted with the approval of the Medical Ethics Committee of Shandong University and informed written consent was received from all subjects. The manuscript does not contain identifying patient information. The data were analyzed anonymously and all clinical investigations were conducted according to the principles expressed in the Declaration of Helsinki.

### Study subjects

Two hundred and thirty patients with GC were recruited in Shandong Province, northeastern China, between January 2011 and December 2012. All diagnoses of GC were pathologically confirmed; exclusion criteria included a history of cancer of any other organ (not originally from stomach) or having undergone radiotherapy or chemotherapy. Three hundred and twenty-eight cancer-free individuals without any detectable or known cancers were collected as controls. All these subjects were living in the same residential areas as the cases, the vast majority of them were selected from the healthy volunteers, and a small portion of our aged controls were collected from inpatients with mild cardiovascular diseases of the hospitals. Their age and sex were matched with those of patients with GC. All subjects were genetically unrelated ethnic Han Chinese. Each subject was evaluated individually with a pretested questionnaire to obtain demographic data and information on related risk factors, including tobacco smoking and alcohol consumption. Individuals who smoked at least once a day for longer than one year were defined as smokers, and those who consumed three or more alcoholic drinks per week for more than six months were considered alcohol drinkers. Clinical data and pathological characteristics of patients were collected and confirmed from their medical history records and questionnaires, and GC tumor, node and metastasis (TNM) stages were classified according to the system of the World Health Organization (WHO).

### Specimens and DNA extraction

One mL peripheral blood sample was collected from each subject. Genomic DNA was isolated from each sample using a modified salt extraction technique [20].

We obtained tissue samples from 36 GC patients in our cohort, and samples from each patient consisted of cancerous tissue, the respective para-carcinoma (defined as being 1.0 cm away from the tumor mass) and surrounding noncancerous gastric mucosal tissues. Genomic DNA was extracted from these samples using the Blood and Cell Culture DNA Mini Kit (Tiangen Biotech, Beijing, China).

### Assessment of allele sizes

*MUC5AC-u* repetitive region genotyping was performed using the polymerase chain reaction (PCR); the gene-specific primer sequences used were as follows: sense 5′- TCCACCCTAACCCTGTCAGCCGC-3′; antisense 5′- GTGGCAGGAGTGTGGGGAAAGG G-3′. PCR amplification of DNA was performed in a total reaction volume of 50 µL, containing 100 ng genomic DNA, 0.2 µM of each primer and 25 µL PrimeSTAR Max DNA Polymerase (Takara, Japan). PCR was conducted in a 9700 Thermacycler (Perkin-Elmer, CA, USA) as follows: a 5 minute initial denaturation at 94 °C, followed by 30 cycles of 10 s at 98°C and 2 minutes at 68°C. PCR products were analyzed by gel electrophoresis (1 volt/cm) in TAE buffer through 1.0% agarose gel.

### DNA sequencing assay

To confirm the genotyping results, PCR-amplified DNA samples (amplicons) were selected and sent to BGI Tech (Beijing, China) for purification and Sanger sequencing. This assay was conducted blind with respect to the specimens and study design.

### Statistical analysis

SPSS 13.0 software (SPSS, Chicago, IL, USA) was employed for statistical analysis. Differences in demographic variables, smoking and drinking habits, and grouped allelic frequencies between case and control participants were compared using the chi-squared test or Fisher's exact test. Regression analyses were performed to determine the odds ratios (ORs) for association of GC and *MUC5AC-u* repetitive region genotypes between the controls and GC patients. ORs were estimated using the natural logarithm and its standard error. The chi-squared test or Fisher's exact test were used for comparison of clinical and pathological characteristics of patients. In order to allow multiple comparisons, p values were corrected (pc) using the Bonferroni correction; pc = p×31 as, across the whole study, 31 statistical tests were conducted. All tests were two-sided, with pc<0.05 considered to be statistically significant.

## Results

### Identification of the *MUC5AC-u* repetitive region

**Figure 1:**
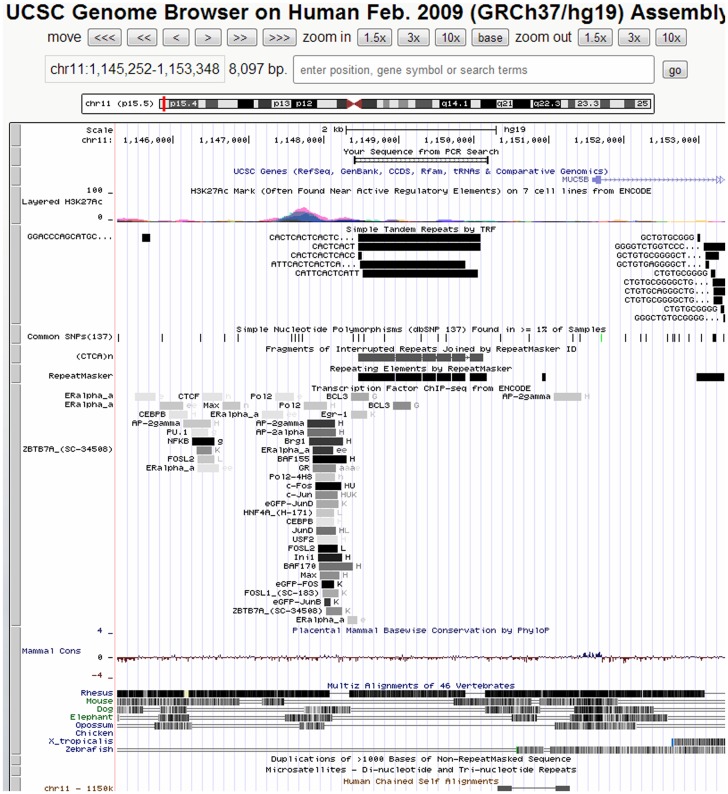
Location and genomic features of the upstream region of the human *MUC5AC* gene. The studied *MUC5AC-u* repetitive region (red and black boxes) is located about 1.5 kb upstream from the *MUC5AC* mRNA transcript start, the *MUC5AC* transcript is incorrectly annotated to *MUC5B* (UCSC Genome Brower, GRCh 37/hg 19). This locus snapshot illustrates its location, the PCR coverage in amplifying the target region (open box with vertical lines), the major repetitive genomic features defined by Repeat Master, the histone H3 lysine 27 acetylation (H3K27AC) enrichment and reported transcription factor binding sites, and many more other features.

Intensive review of the *MUC5AC* upstream region identified a complicated 1710 bp repetitive region (termed the *MUC5AC-u* repetitive region) located between nucleotides −3162 to −1452 upstream from the ATG initiation codon (Figure 1). This position is immediately downstream of a genomic locus with the capacity to bind several transcription factors. The *MUC5AC-u* repetitive region contains many interrupted irregular repeats of different lengths and is a complicated combination of microsatellite (e.g., CTCA), minisatellite (e.g., CATTCACT or CATTCACTCATT) and megasatellite (e.g., ACCCATTCACTCACTCACTTATTCACTC) repeats. At the 5′ region, a 300 bp sequence was found to be duplicated exactly, head-to-tail.

### Study population

All individuals in the study (328 cancer-free controls and 230 GC patients) were from a Han Chinese population and without any known hereditary disease. Both groups had similar distributions of age, sex and alcohol consumption (χ^2^ test; p = 0.875, p = 0.589, p = 0.770, respectively; Table S1). There was no significant difference in the distribution of cigarette smoking between the patients and controls (p = 0.098). According to the TNM system, 10.9%, 10.0%, 21.7%, 42.2% and 15.2% of patients had stage 0, I, II, III and IV disease, respectively (Table S2).

### Association of repetitive region genotypes with the risk of GC

**Figure 2:**
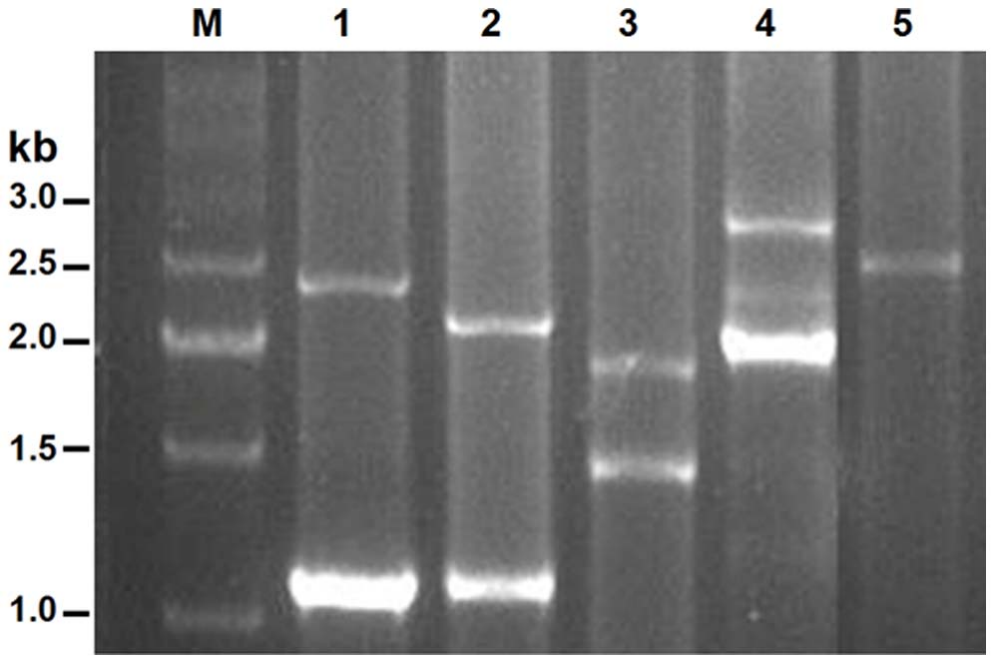
Representative alleles of *MUC5AC-u* repetitive region. *MUC5AC-u* repetitive regions were PCR-amplified from blood genomic DNA of case and control samples using specific unique primers. Eight alleles with discontinuous sizes ranging from 1.1 to 2.8 kb were identified in this Han Chinese population. Lane 1: 1.1 kb/2.3 kb; lane 2: 1.1 kb/2.1 kb; lane 3: 1.4 kb/1.8 kb; lane 4: 2.0 kb/2.8 kb; lane 5: 2.5 kb/2.5 kb. M indicated the size marker.

Genomic DNA samples were isolated from whole blood of all the subjects and used as templates to amplify the *MUC5AC-u* repetitive region. Eight alleles with discontinuous sizes ranging from 1.1 to 2.8 kb were identified in this Han Chinese population (Figure 2). The 1.1 kb allele was most common, the 1.8 and 2.0 kb alleles were less common, and the others were all relatively uncommon (Table 1).

**Table 1:** Distribution of *MUC5AC-u* repetitive region alleles among cases and controls.

Allele size	n (%)		Cases *vs.* controls			
(kb)	Cases (n = 460)	Controls (n = 656)	OR	95% CI	p	pc
**1.1**	197 (42.8)	307 (46.8)	0.852	0.670–1.083	0.189	
**1.4**	18 (3.9)	0 (0.0)	-	-	**9.67×10^−8^**	**3.00×10^−6^**
**1.8**	163 (35.4)	169 (25.8)	1.582	1.221–2.049	**5.03×10^−4^**	**1.56×10^−2^**
**2.0**	50 (10.9)	78 (11.9)	0.904	0.620–1.317	0.598	
**2.1**	5 (1.1)	6 (0.9)	1.190	0.361–3.924	0.774	
**2.3**	15 (3.3)	59 (9.0)	0.341	0.191–0.609	**1.51×10** ^−**4**^	**4.68×10^−3^**
**2.5**	12 (2.6)	25 (3.8)	0.676	0.336–1.360	0.269	
**2.8**	0 (0.0)	12 (1.8)	-	-	**0.002**	**0.062**

pc, p value corrected using the Bonferroni correction for multiple comparisons. 31 statistical tests were totally conducted in the whole study, and pc = p×31.

The overall distribution of the *MUC5AC-u* repetitive region alleles among patients with GC differed significantly from that found in controls (χ^2^ = 58.44, p = 3.09×10^−10^). For further analysis, comparisons of allele frequencies between patients and controls were made individually for each allele, using Fisher's test (Table 1). The 1.4 and 1.8 kb alleles were significantly more prevalent in patients with cancer than in controls (3.9% *vs.* 0.0%, pc = 3.00×10^−6^; 35.4% vs. 25.8%, pc = 1.56×10^−2^, respectively). Additionally, the frequencies of the 2.3 and 2.8 kb alleles were significantly lower in patients with cancer than in controls (3.3% vs. 9.0%, p = 1.51×10^−4^; 0.0% vs. 1.8%, p = 0.002, respectively), and the multiple comparisons corrected p values were 4.68×10^−3^ for the 2.3 kb and 0.062 (suggestive) for the 2.8 kb allele. No significant differences were found when frequencies of other alleles between cases and controls were compared.

Based on these observations, we classified the eight alleles as susceptible (S), protective (P), or null with respect to risk (N) as follows: S, 1.4 or 1.8 kb; P, 2.3 or 2.8 kb; and N, all other alleles. Twenty-one *MUC5AC-u* repetitive region genotypes were totally identified in our case-control population (Table S3), the genotypes were then defined as NN, SN, PN, SP, SS, and there was no PP genotype in our cohort. The most common genotype (NN) was designated as the reference group. Individuals with the homozygous genotype SS had a 2.7-fold increased risk of GC occurrence (OR = 2.683, 95% CI = 1.554–4.361, pc = 0.012; Table 2). The PN genotype was associated with a significantly reduced risk of GC (OR = 0.257, 95% CI = 0.116–0.569, pc = 0.031). Neither of the heterozygous genotypes SN and SP was associated with a change in the risk of GC (both p>0.05).

**Table 2:** Association of *MUC5AC*-u repetitive region genotypes and gastric cancer risk.

Genotypes	n (%)			Cases *vs.* controls			
	Cases (n = 230)	Controls (n = 328)	Total (n = 558)	OR	95% CI	p	pc
**NN**	87 (37.8)	137 (41.8)	224 (40.1)	1			
**SS**	46 (20.0)	27 (8.2)	73 (13.1)	**2.683**	**1.554–4.631**	**3.95×10^−4^**	**1.22×10^−2^**
**SP**	7 (3.0)	22 (6.7)	29 (5.2)	0.501	0.205–1.222	0.129	
**SN**	82 (35.7)	93 (28.4)	175 (31.4)	1.388	0.930–2.072	0.108	
**PN**	8 (3.5)	49 (14.9)	57 (10.2)	**0.257**	**0.116–0.569**	**0.001**	**0.031**

pc, p value corrected using the Bonferroni correction for multiple comparisons. 31 statistical tests were totally conducted in the whole study, and pc = p×31.

### Clinical and pathological characteristics at diagnosis of GC patients with differing *MUC5AC-u* repetitive regions

As certain variable number of tandem repeat polymorphisms are reported to exert dual, conflicting effects on the risk and prognosis of cancer [21], we compared the age at onset and clinical stages between GC patients with and without *MUC5AC-u* repetitive regions of 1.4, 1.8 or 2.3 kb separately.

In our sample, fifteen GC patients (6.5%) carried the 1.4 kb allele; three of them were homozygous for this allele and the remainder were heterozygous. Significantly higher percentages of GC patients with at least one copy of the 1.4 kb allele were younger (<50 years) individuals or with more advanced T (T4) and M (M1) stages compared with those lacking it (66.7% vs. 17.2%, p = 4.37×10^−6^; 93.3% vs. 58.6%, p = 0.006; 53.3% vs. 12.6%, p = 2.13×10^−5^, respectively; pc values  = 1.35×10^−4^, 0.186 and 6.60×10^−4^, respectively, after correcting for multiple comparisons; Table 3).

**Table 3:** Clinical and pathological characteristics of GC patients with *MUC5AC-u* repetitive region 1.4 kb allele.

	1.4 kb/1.4 kb+1.4 kb/non-1.4 kb n (%)	non-1.4kb/non-1.4 kb n (%)	p	pc
**Total cases**	15 (6.5)	215 (93.5)		
**Age (years)**				
<50	10 (66.7)	37 (17.2)	**4.37×10^−6^**	**1.35×10^−4^**
≥ 50	5 (33.3)	178 (82.8)		
**T stage**				
Tis-T3	1 (6.7)	89 (41.4)	**0.006**	0.186
T4	14 (93.3)	126 (58.6)		
**N stage**				
N0	5 (33.3)	70 (32.6)	0.951	
N1-3	10 (66.7)	145 (67.4)		
**M stage**				
M0	7 (46.7)	188 (87.4)	**2.13×10^−5^**	**6.60×10^−4^**
M1	8 (53.3)	27 (12.6)		
**TNM stage**				
Stage 0–II	3 (20.0)	95 (44.2)	0.103	
Stage III–IV	12 (80.0)	120 (55.8)		

pc, p value corrected using the Bonferroni correction for multiple comparisons. 31 statistical tests were totally conducted in the whole study, and pc = p×31.

There were 128 GC patients (55.7%) in our sample who carried the 1.8 kb version of the *MUC5AC-u* repetitive region; 35 patients were homozygous for this allele. Homozygous patients tended to have an older age of onset (≥ 50 years), and less advanced T (Tis-T3), N (N0), and TNM (stage 0–II) stages compared with patients who were not homozygous for the 1.8 kb allele (5.7% vs. 23.1%, p = 0.021; 60.0% vs. 35.4%, p = 0.006; 51.4% vs. 29.2%, p = 0.010; 68.6% vs. 37.9%, p = 7.43×10^−4^, respectively), although most of the nominally significant p values did not survive the Bonferroni correction (Table 4).

**Table 4:** Clinical and pathological characteristics of GC patients with *MUC5AC-u* repetitive region 1.8 kb allele.

	1.8 kb/1.8 kb n (%)	1.8 kb/non-1.8 kb+ non-1.8 kb/non-1.8 kb n (%)	p	pc
**Total cases**	35 (15.2)	195 (84.8)		
**Age (years)**				
<50	2 (5.7)	45 (23.1)	**0.021**	0.651
≥50	33 (94.3)	150 (76.9)		
**T stage**				
Tis-T3	21 (60.0)	69 (35.4)	**0.006**	0.186
T4	14 (40.0)	126 (64.6)		
**N stage**				
N0	18 (51.4)	57 (29.2)	**0.010**	0.31
N1-3	17 (48.6)	138 (70.8)		
**M stage**				
M0	33 (94.3)	162 (83.1)	0.124	
M1	2 (5.7)	33 (16.9)		
**TNM stage**				
Stage 0–II	24 (68.6)	74 (37.9)	**7.43×10^−4^**	**0.023**
Stage III–IV	11 (31.4)	121 (62.1)		

pc, p value corrected using the Bonferroni correction for multiple comparisons. 31 statistical tests were totally conducted in the whole study, and pc = p×31.

We did not find individuals showing the homozygous genotype 2.3/2.3 kb in our sample; however, fifteen GC patients (6.5%) were heterozygous for this allele. Heterozygous patients were older at GC onset than patients who were not, although this result was at a marginal level of significance and did not survive the correction for multiple tests. There was no significant difference in distributions of T, N, M or TNM stages of cancer between patients with one or no copy of the 2.3 kb allele (Table 5).

**Table 5:** Clinical and pathological characteristics of GC patients with *MUC5AC-u* repetitive region 2.3 kb allele.

	2.3 kb/non-2.3 kb n (%)	non-2.3 kb/non-2.3 kb n (%)	p	pc
**Total cases**	15 (6.5)	215 (93.5)		
**Age (years)**				
<50	0 (0.0)	47 (21.9)	0.045	1.000
≥50	15 (100.0)	168 (78.1)		
**T stage**				
Tis-T3	6 (40.0)	84 (39.1)	0.943	
T4	9 (60.0)	131 (60.9)		
**N stage**				
N0	3 (20.0)	72 (33.5)	0.396	
N1-3	12 (80.0)	143 (66.5)		
**M stage**				
M0	13 (86.7)	182 (84.7)	1.000	
M1	2 (13.3)	33 (15.3)		
**TNM stage**				
Stage 0–II	5 (33.3)	93 (43.3)	0.452	
Stage III–IV	10 (66.7)	122 (56.7)		

pc, p value corrected using the Bonferroni correction for multiple comparisons. 31 statistical tests were totally conducted in the whole study, and pc = p×31.

### Analysis of repetitive region instability in cancer tissues

**Figure 3:**
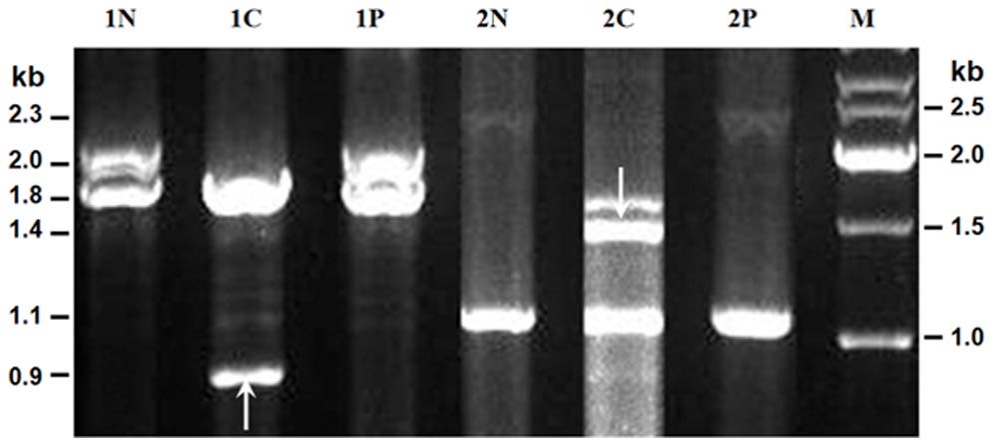
Instability of *MUC5AC-u* repetitive region in normal, para-carcinoma, and cancer tissues from patients with gastric cancers. Genomic DNA was analyzed from cancer, para-carcinoma and surrounding normal tissues of patients. The sizes of *MUC5AC-u* repetitive region were analyzed by PCR. N indicated gastric normal tissues, C indicated cancer tissues, P indicated para-carcinoma tissues, and M indicated the size marker. Rearrangements in cancer tissues are indicated by arrows. Heterozygotes have an additional hetero-duplex band (lane of 2C).

As repetitive regions of DNA are unstable in various human malignancies, including GC [22], we next determined whether the hypervariable *MUC5AC-u* repetitive regions differed in length between cancer, para-carcinoma and surrounding normal tissues from 36 GC patients. The results showed no differences in band pattern between para-carcinoma and normal tissues in all 36 patients; however, length alterations were observed in DNA samples of cancer tissues in two GC patients (Figure 3). In both cases, bands were detected showing a shift from long alleles in cancer tissue to short alleles in para-carcinoma tissue. In one case, one allele shifted from 2.0 kb to a novel, 0.9 kb allele, and, in another case, one allele shifted from 2.3 kb to 1.4 kb. Among the 36 gastric cancer patients tested, the frequency of cancer-related genome rearrangement in the *MUC5AC-u* repetitive region was 5.6%.

### Sanger sequencing of the 1.1, 1.4 and 1.8 kb alleles from three GC patients

PCR amplicons of the 1.1 kb and 1.4 kb alleles from the gastric cancer tissue DNA were successfully sequenced using the Sanger sequencing technique. These sequences are listed in supporting information files. We were unable to sequence the entire fragment of a 1.8 kb amplicon (PCR amplicon using the gastric cancer tissue DNA), or any other fragments >1.8 kb, due to the complicated and repetitive structure of the target region and limitations of the technique. The sequences show the same main genetic structure and repetitive units as the UCSC genome reference sequence but with different overall lengths. The initial 300 bp at the 5' end of the 1.4 kb *MUC5AC-u* repetitive region sequence are exactly duplicated in a head-to-tail pattern.

## Discussion

In this study, we assessed the association of genetic variation in a repetitive region close to the *MUC5AC* promoter with the risk of occurrence and progression of GC. Our study was suggested by the diverse biological functions of MUC5AC in the healthy and diseased states, the unique location of the region potential regulating the gene expression, the highly dynamic nature of the repetitive sequence, and the effect of this instability on generating novel mutations.

Analysis of 230 GC patients and 328 controls showed the *MUC5AC-u* repetitive region was highly polymorphic, with eight different alleles (plus a 0.9 kb allele in the cancer tissue from one GC patient) being present in a Han Chinese population from northeastern China. Based on the distribution and differences of allelic frequencies between GC patients and controls, these eight alleles were classified into susceptible alleles (S: 1.4 and 1.8 kb), protective alleles (P: 2.3 and 2.8 kb), and null alleles (N: the others). Individuals bearing two susceptible alleles (SS) had a 2.7-fold increased risk of developing GC, and the genotype PN was associated with a reduced risk of gastric cancer. Our findings suggest that genetic variation in this region is significantly associated with susceptibility to GC, and thus add to the existing evidence that changes in MUC5AC expression is involved in the pathogenesis of this malignant disease.

In further analysis, we found that these genetic variants were not only associated with GC susceptibility, but also with its prognosis. We found patients with the 1.4 kb allele had an earlier age of GC onset and were more likely to have advanced T and M stage diseases. As advanced T and M stages are associated with a poor prognosis in general, our results indicated that GC patients with the 1.4 kb allele were linked to more rapid progression of the disease. In contrast, patients homozygous for the 1.8 kb allele tended to have an older age at diagnosis and less advanced T, N, and TNM stages than other patients, indicating this genotype might decrease the risk of developing advanced gastric cancer and be associated with a better outcome.

Repetitive regions of the genome have been dismissed as nonfunctional “junk” DNA previously; however, a recent study found that up to 25% of gene promoters in the *Saccharomyces cerevisiae* genome contain repetitive sequences [23]. A comparable distribution of tandem repeats in the promoters of *Homo sapiens* genes also demonstrated that genes driven by repeat-containing promoters had significantly higher rates of transcriptional divergence [23]. A number of studies have shown that many variations in repetitive regions of promoters affect gene expression and contribute to genetic susceptibility to various human disorders [24]–[26], and for cancers as well [27]–[29]. Several molecular mechanisms may underlie the effects of repetitive regions in promoters on gene expression; for example, they may alter the number of transcription factor binding sites, generate changes in the spacing of critical promoter elements, modulate the activity of RNA-binding proteins or affect the chromatin structure [23]. According to the Encyclopedia of DNA Elements (ENCODE) dataset, available for visualization and download via the UCSC Genome Browser (http://genome.ucsc.edu/), the region containing the *MUC5AC-u* repetitive region contains clusters of known transcription factor binding sites and is enriched for histone H3 lysine 27 acetylation (H3K27ac), a reliable marker for active chromatin. Thus length variations of this repetitive region might have considerable impact on DNA structure and transcription factor binding, and hence upon gene regulation. Therefore, our finding of an association between the length of the repetitive region and a change in GC risk might be explained by alterations in MUC5AC levels. This will be explored in future studies, which will investigate whether the 1.4 and 1.8 kb alleles enhance promoter activity and if 2.3 and 2.8 kb alleles repress it. Such studies will help to reveal the exact role this region plays in the development and prognosis of GC.

Genomic instability was shown to affect tumor initiation and progression by accelerating the accumulation of the multiple genetic alterations responsible for cancer cell development [30]. Although spontaneous rearrangements of repetitive regions were detected more frequently in the germ line than in somatic cells [31], several studies have demonstrated repetitive regions are unstable in various human neoplasms [32]–[34], including GC [35]. When we examined the *MUC5AC-u* repetitive region length in DNA from normal and cancer tissues from some GC patients, we found two examples of length alterations. Both converted long to short alleles, and the 1.4 kb allele, associated in our study with an increased risk of GC, appeared in one case. Although the genetic rearrangement frequency was relatively low, this result implies that instability at this locus contributes to the pathogenesis of gastric cancer in some cases.

The duplication of a 300 bp DNA segment at the beginning of this complicated repetitive region is relevant in this context. This duplication is likely to have occurred multiple times to form the larger allelic variants, which differ from each other mostly in ∼300 bp increments. For example, we speculate the 1.4 kb allele was generated by this duplication from the 1.1 kb allele, which is the most common allele in our study population. This duplication event may be associated with genome instability which is extensively involved in tumorigenesis, development and metastasis of gastric cancer. There are reports of similar duplication events in large central repetitive exons of *MUC5AC* [36].

Although we did not achieve novel sequences distinguishing them with the reference sequence in three selected GC patients, from 1.1, 1.4 and 1.8 kb allele DNA fragments, we found many SNPs, which could possibly be used as proxies for allele sizes and in strong LD with other genetic markers outside of the region, besides of the length differences. Due to the great complexity, length, high similarity across the region, and the limits of the sequencing technique, we could not sequence the entire region of the DNA amplicons from all subjects; thus, other sequencing features and genetic variants have not been revealed very likely. Moreover, we can not tell if there are more dramatic genetic mutation events occurred in the genome DNA of the cancer tissue which will be more challenging, but likely more productive.

To the best of our knowledge, this is the first report to indicate the association between genetic variation in *MUC5AC-u* repetitive region and gastric cancer risk. We have shown certain genetic length variants in the repetitive region around the *MUC5AC* promoter are significantly associated with susceptibility to GC, and with its clinical stages. Prospective, large-scale trials, as well as well-designed mechanistic studies, are required to validate our findings.

## Supporting Information

Figure S1 Multiple sequence alignment of MUC5AC-u repetitive region variants. PCR amplicons of the 1.1 kb, 1.4 kb, and 1.8 kb alleles from the gastric cancer tissue DNA were sequenced using the Sanger sequencing technique. A.1.1 kb full sequence. B. 1.4 kb full sequence. C. 1.8 kb allele sequence with a gap at 3′ side.
(TIF)

Table S1 Distributions of selected characteristics in gastric cancer cases and controls.
(DOC)

Table S2 TNM stages in cases of gastric cancer.
(DOC)

Table S3 Distribution of MUC5AC-u repetitive region genotypes in gastric cancer cases and controls.
(DOC)
