# Supplementary figures and images for: MUC5AC Upstream Complex Repetitive Region Length Polymorphisms Are Associated with Susceptibility and Clinical Stage of Gastric Cancer
Source: PLoS One. 2014 Jun 2;9(6):e98327. doi: 10.1371/journal.pone.0098327 (PMC4041751; doi:10.1371/journal.pone.0098327)

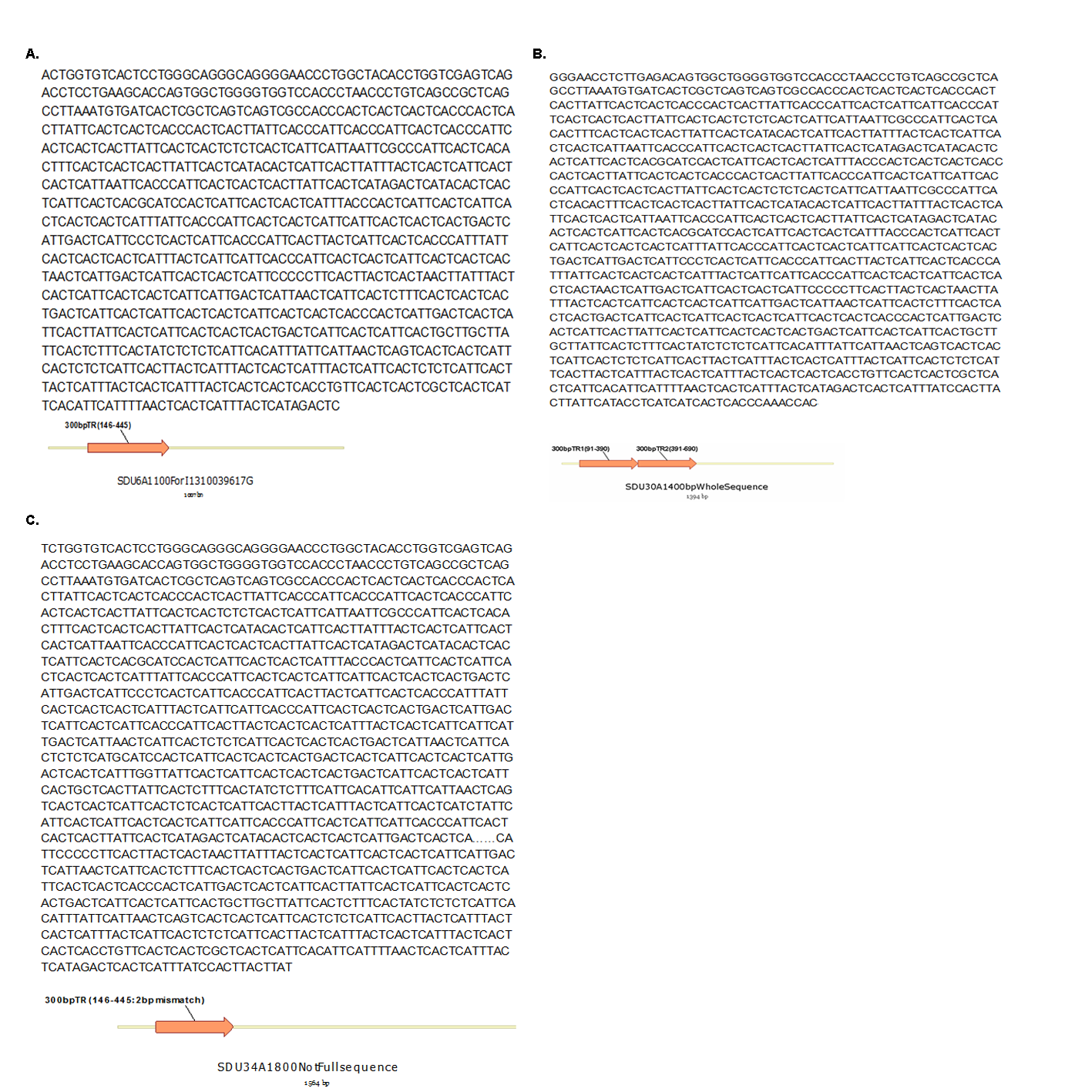

Supplement: Figure S1 — Multiple sequence alignment of MUC5AC-u repetitive region variants. PCR amplicons of the 1.1 kb, 1.4 kb, and 1.8 kb alleles from the gastric cancer tissue DNA were sequenced using the Sanger sequencing technique. A.1.1 kb full sequence. B. 1.4 kb full sequence. C. 1.8 kb allele sequence with a gap at 3′ side. (TIF) [file pone.0098327.s001.tif]
