# Supplementary material for: MUC5AC Upstream Complex Repetitive Region Length Polymorphisms Are Associated with Susceptibility and Clinical Stage of Gastric Cancer
Source: PLoS One. 2014 Jun 2;9(6):e98327. doi: 10.1371/journal.pone.0098327 (PMC4041751; doi:10.1371/journal.pone.0098327)
Supplement: Table S1 — Distributions of selected characteristics in gastric cancer cases and controls. (DOC) [file pone.0098327.s002.doc]

Table S1. Distributions of selected characteristics in gastric cancer cases and controls

| **Variable** | **Level** | **Controls, [n=328] n (%)** | **Cases, [n=230] n (%)** | **P** | **χ^2^-value** |
| --- | --- | --- | --- | --- | --- |
| Age (y) | 30–49 | 61 (18.6%) | 47 (20.4%) | 0.875 | 1.222 |
|  | 50–59 | 75 (22.9%) | 55 (23.9%) |  |  |
|  | 60–69 | 125 (38.1%) | 82 (35.7%) |  |  |
|  | 70–79 | 66 (20.1%) | 46 (20.0%) |  |  |
|  | 80+ | 1 (0.3%) | 0 (0.0%) |  |  |
| Sex | Women | 111 (33.8%) | 83 (36.1%) | 0.589 | 0.301 |
|  | Men | 217 (66.2%) | 147 (63.9%) |  |  |
| Smoking | No | 150 (45.7%) | 89 (38.7%) | 0.098 | 2.733 |
|  | Yes | 178 (54.3%) | 141 (61.3%) |  |  |
| Drinking | No | 177 (54.0%) | 127 (55.2%) | 0.770 | 0.086 |
|  | Yes | 151 (46.0%) | 103 (44.8%) |  |  |
