# Supplementary material for: MUC5AC Upstream Complex Repetitive Region Length Polymorphisms Are Associated with Susceptibility and Clinical Stage of Gastric Cancer
Source: PLoS One. 2014 Jun 2;9(6):e98327. doi: 10.1371/journal.pone.0098327 (PMC4041751; doi:10.1371/journal.pone.0098327)
Supplement: Table S2 — TNM stages in cases of gastric cancer. (DOC) [file pone.0098327.s003.doc]

Table S2. TNM stages in cases of gastric cancer

| **TNM Stage** | **n (%)** |
| --- | --- |
| 0 | 25 (10.9) |
| I | 23 (10.0) |
| II | 50 (21.7) |
| III | 97 (42.2) |
| IV | 35 (15.2) |
| Total | 230 (100.0) |
