# Supplementary material for: MUC5AC Upstream Complex Repetitive Region Length Polymorphisms Are Associated with Susceptibility and Clinical Stage of Gastric Cancer
Source: PLoS One. 2014 Jun 2;9(6):e98327. doi: 10.1371/journal.pone.0098327 (PMC4041751; doi:10.1371/journal.pone.0098327)
Supplement: Table S3 — Distribution of MUC5AC-u repetitive region genotypes in gastric cancer cases and controls. (DOC) [file pone.0098327.s004.doc]

**Table S3. Distribution of *MUC5AC*-*u*** repetitive region genotypes in gastric cancer cases and controls

| **Genotype** | **SPN classification** | **Cases**  **n (%)** | **Control**  **n (%)** |
| --- | --- | --- | --- |
| 1.1/1.1 | NN | 57 (24.8) | 90 (27.4) |
| 1.1/1.8 | SN | 52 (22.6) | 72 (22.0) |
| 1.8/1.8 | SS | 35 (15.2) | 27 (8.2) |
| 1.1/2.0 | NN | 18 (7.8) | 30 (9.1) |
| 1.8/2.0 | SN | 22 (9.6) | 10 (3.0) |
| 1.1/1.4 | SN | 2 (0.9) | 0 (0.0) |
| 1.1/2.1 | NN | 0 (0.0) | 6 (1.8) |
| 1.1/2.3 | PN | 5 (2.2) | 19 (5.8) |
| 1.1/2.5 | NN | 6 (2.6) | 0 (0.0) |
| 1.4/1.4 | SS | 3 (1.3) | 0 (0.0) |
| 1.4/1.8 | SS | 8 (3.5) | 0 (0.0) |
| 1.4/2.0 | SN | 2 (0.9) | 0 (0.0) |
| 1.8/2.1 | SN | 2 (0.9) | 0 (0.0) |
| 1.8/2.3 | SP | 7 (3.0) | 17 (5.2) |
| 1.8/2.5 | SN | 2 (0.9) | 11 (3.4) |
| 1.8/2.8 | SP | 0 (0.0) | 5 (1.5) |
| 2.0/2.0 | NN | 4 (1.7) | 4 (1.2) |
| 2.1/2.3 | PN | 3 (1.3) | 0 (0.0) |
| 2.5/2.5 | NN | 2 (0.9) | 7 (2.1) |
| 2.0/2.3 | PN | 0 (0.0) | 23 (7.0) |
| 2.0/2.8 | PN | 0 (0.0) | 7 (2.1) |
